# Floristic assessment and soil-vegetation dynamics in an arid zone: a case study of the old Katameya-Ain Sokhna Road, Eastern Desert, Egypt

**DOI:** 10.1038/s41598-025-22507-z

**Published:** 2025-10-29

**Authors:** Shaimaa G. Salama, A. H. Marie, Ramadan Bedair

**Affiliations:** 1 Botany and Microbiology Department, Faculty of Science, Damanhour University, Damanhour, 22516 Egypt; 2 Botany and Microbiology Department, Faculty of Science, Al- Azhar University, Nasr City, 11884 Cairo Egypt

**Keywords:** Plant diversity, Vegetation-soil relationship, Conservation status, Anthropogenic impacts, SAVI, DCA, Medicinal plants, Eastern desert, Egypt, Ecology, Ecology, Environmental sciences, Plant sciences

## Abstract

In the hyper-arid deserts of Egypt, where extreme environmental conditions prevail, understanding the symbiotic relationship between the sparse vegetation cover and the underlying soil is crucial for unraveling the mechanisms of plant survival and ecosystem functioning. This study investigates vegetation composition, environmental drivers, and their interactions in an unprotected area of Egypt’s northern Eastern Desert, which has recently experienced substantial anthropogenic disturbances. Primary threats to vegetation include road construction, unregulated quarrying, and overexploitation of natural resources. To represent the habitats present in the study area, 20 stands were selected. In each stand, the existing plant species were recorded, soil samples were taken from each stand, and multivariate statistics (DCA) were conducted to show the relationship between the distinctive plant indicators for each stand, as well as the most influential soil factors in each stand. Then, the state of the vegetation cover in previous years (2014) was compared with the state in the study year (2024). A total of 75 plant species were documented, with Asteraceae (15 species) and Fabaceae (6 species) representing the most diverse families. Perennials (73%), chamaephytes (44%), and Saharo-Arabian species (71%) dominated the flora. Detrended Correspondence Analysis (DCA) revealed that the studied stands were divided into four groups (A, B, C, and D). Each group was ecologically similar to the other. Each group of stands had distinctive plant indicators and the soil factors most closely associated with them. Based on the IUCN Red List, conservation status assessments were provided for each species (65 species were unevaluated, while only 11% of the total species are classified as Least Concern, with no taxa appearing in the threatened category). The presence of invasive non-native taxa, such as *Beta vulgaris* and *Centaurea calcitrapa*, which threaten native biodiversity, was noted. Analysis of the Soil Adjusted Vegetation Index (SAVI) revealed a reduction in vegetation cover between 2014 (SAVI range: -0.523911 to 0.860437) and 2024 (SAVI range: -0.574714 to 1.08698). The recorded plant species include 16 medicinal plants. Escalating habitat destruction and anthropogenic pressures underscore the urgent need for targeted conservation strategies to safeguard biodiversity in this vulnerable region.

**Supplementary Information:**

The online version contains supplementary material available at 10.1038/s41598-025-22507-z.

## Introduction

Arid ecosystems, defined by scarce water resources, pose distinct challenges and opportunities for plant survival, driving specific adaptations and ecological interactions critical for ecosystem function^1^. Projected global increases in aridity due to climate change are anticipated to alter the structure and dynamics of dryland ecosystems^2^. The delicate equilibrium among these regions’ soil properties, vegetation patterns, and floristic composition underpins ecosystem stability and resilience^3^. Detailed analyses of these factors can elucidate key ecological mechanisms governing plant establishment, growth, and spatial distribution in water-limited habitats^4^. Egypt’s extensive desert landscapes represent a quintessential arid environment, where soil-vegetation dynamics and floristic diversity studies hold particular significance for ecological understanding^5^. However, human activities and centralized governance often destabilize traditional land-use systems, accelerating desertification processes^6^. Pressures from rural livelihoods may intensify the overexploitation of natural resources, further driving environmental decline^7^.

Deciphering the complex linkages between soil attributes, plant community structure, and species assemblages is vital for developing targeted conservation approaches and sustainable land-use frameworks in these vulnerable ecosystems. Such insights advance knowledge of plant-environment interactions in arid zones, offering actionable data for ecological rehabilitation and resource management strategies^8^. Despite these harsh conditions, the flora of these ecosystems exhibits remarkable resilience, contributing to soil stabilization, nutrient cycling, and microhabitat creation for associated fauna^9^. The ecological importance of Wadi plants extends to their role as genetic reservoirs for drought and heat tolerance, offering insights into adaptive mechanisms under climate change scenarios^10^. Additionally, these species support local biodiversity by serving as keystone resources for pollinators and herbivores, which are integral to desert food webs^11^. Ethnobotanical studies further highlight their socio-economic value, as many species are utilized by indigenous communities for traditional medicine, fodder, and fuel, underscoring the interdependence between human livelihoods and plant conservation^12,13^. However, climate change as well as increasing anthropogenic pressures, such as overgrazing and habitat fragmentation, threaten this biodiversity hotspot, necessitating urgent conservation strategies informed by ecological studies^14,15^.

Plant diversity in the Wadis of the Eastern Desert of Egypt represents a critical component of arid ecosystems, sustaining ecological functions and providing unique adaptations to extreme environmental conditions. Research on the flora and plant diversity of the Eastern Desert of Egypt has evolved over decades, addressing ecological patterns and anthropogenic impacts. Early phytosociological studies, such as those in Wadi Hagul by Mashaly^16^, established foundational knowledge on plant community structure in hyper-arid environments. Subsequent work by Salama et al.^17^ expanded this understanding by analyzing floristic composition across distinct Wadis, including Wadi Al-Assiuty and Wadi Habib. Abdelaal^18^ studied Wadi Hagul to assess temporal changes in floristic diversity, identifying declines in native species richness attributed to habitat degradation. More recently, Bedair et al.^19^ quantified the effects of human disturbance in Wadi Hagul, demonstrating how quarrying and unregulated grazing alter vegetation dynamics and reduce ecosystem resilience. Together, these studies underscore the interplay of ecological factors shaping plant diversity in Egypt’s Eastern Desert while highlighting the conservation status of recorded plant species. The present study investigates the floristic composition, conservation status of plant species, and edaphic characteristics within the old Katameya-Ain Sokhna Road, Eastern Desert, Egypt, employing an integrated approach to assess vegetation-soil interactions through multivariate analysis.

## Materials and methods

### Floristic composition

All stands in the study area were surveyed during an initial field trip. First, the minimal area of the quadrat in the study area was determined following standard phytosociological practice. Twenty stands were then selected; in each, four quadrats, each 100 square meters in area, were selected.to represent the major vegetation types, environmental gradients, and ecologically significant habitats in the study area, enabling us to identify all plant species in the various habitats. This approach ensured that both common and rare habitats were adequately represented, which might not have been achieved through random sampling. The plant samples inside each quadrate are also identified, their names, numbers, and percentages are recorded, and samples are taken to the laboratory to confirm their identification, in addition to many other field data. A soil sample is also taken from each quadrat.

**Fig. 1:**
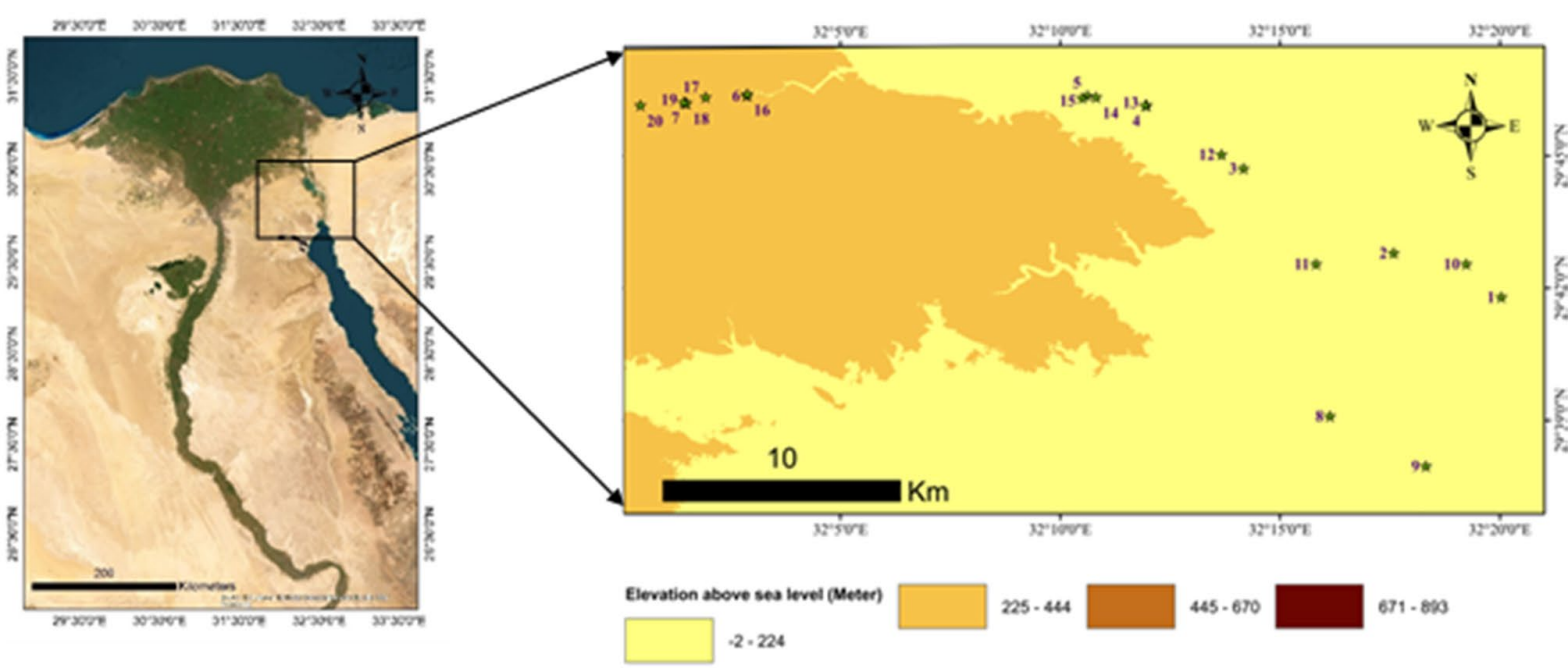
Location on the map showing the studied stands and the digital elevation model of the old Katameya-Ain Sokhna Road (maps created using ArcGIS 10.5).

To assess the floristic composition and vegetation cover along the old Katameya-Ain Sokhna Road, 20 stands were selected during spring 2024 to represent diverse plant habitats in the area (Fig. 1). The website https://portal.opentopography.org/raster?opentopoID=OTSRTM.082015.4326.1 was used to download the digital elevation model (DEM) of the study area. The maps containing the studied stands as well as the elevations in the study area (digital elevation model) was created using ArcGIS 10.5 program (Esri. (2016). ArcGIS Desktop: Release 10.5 [Software]. Environmental Systems Research Institute. https://www.esri.com).

Four quadrats (10 m × 10 m; 100 m² each) were established within each stand. The importance value for each recorded plant species was calculated as a composite measure integrating four key ecological parameters: relative density, relative abundance, relative frequency, and relative cover^20^. This metric quantitatively assesses a species’ ecological dominance and spatial contribution within the vegetation community. and all plant species within the quadrats were identified. Recorded plant species in all studied stands were identified according to Boulos^21–26^. https://powo.science.kew.org/ has updated the scientific names of the recorded species. Species were classified by life span following Boulos^21–25^, while life form and floristic categories were determined using criteria outlined by Raunkiaer^27^, Tutin et al.^28^, and Davis^29^. The conservation status of plant species was determined according to the International Union for Conservation of Nature^30^. Alien species were determined according to El-Beheiry et al.^31^. Invasive species were determined according to^32^.

### Soil analyses

Soil characteristics were evaluated through the analysis of 15 physicochemical parameters: texture (sand, silt, clay), pH, electrical conductivity (EC), total dissolved salts (TDS), organic matter content, saturation percentage, sodium absorption ratio (SAR), and concentrations of calcium (Ca²⁺), magnesium (Mg²⁺), sodium (Na⁺), potassium (K⁺), chloride (Cl⁻), bicarbonate (HCO₃⁻), carbonate (CO₃²⁻), and sulfate (SO₄²⁻). Four soil samples were collected and homogenized for each stand to form a composite sample.

Soil texture was analyzed via the pipette method^33^. Portable digital meters (Adwa^®^ AD8000 series, Szeged, Hungary) were used to measure pH, TDS, and EC. Calcium and magnesium concentrations were determined by titration^34^, while sodium and potassium were quantified using flame photometry [589 nm for Na⁺, 767 nm for K⁺;^33^. Chloride, bicarbonate, and carbonate levels were assessed via titration^35^, and sulfates were measured turbidimetrically^33^. Organic matter content was determined using the Walkley-Black titration method^36^, and SAR was calculated according to Ayers & Westcot^37^. A standardized laboratory protocol determined the soil saturation percentage^38^.

### Statistical analysis

Descriptive statistics, including the range, minimum, maximum, mean, and standard deviation, were calculated for various soil parameters using SigmaPlot software (version 12.5). Detrended Correspondence Analysis (DCA), a multivariate statistical method, was performed using PC-ORD version 5 software to assess the relationships between indicator plant species and edaphic factors associated with the distribution of stands.

### Vegetation change analysis using the soil adjusted vegetation index (SAVI)

To assess changes in vegetation cover within the study area, the Soil-Adjusted Vegetation Index (SAVI) was computed for the years 2014 and 2024. Landsat 8 satellite imagery from the spring season was acquired via the USGS Earth Explorer platform^39^, utilizing Band 4 (red spectrum, 0.64–0.67 μm) and Band 5 (near-infrared spectrum, 0.85–0.88 μm). The images were entered into the ArcGIS 10.5 program SAVI was calculated using the formula:

$${\mathrm{SAVI}}=[({\text{Band 5}}--{\text{Band 4}})/({\text{Band 5}}+{\text{Band 4}}+{\mathrm{L}})] \times ({\mathrm{1}}+{\mathrm{L}}),$$

where **L** represents a soil brightness correction factor, set to 0.5 to account for moderate vegetation cover. Following index computation, the study area boundary was extracted from the processed imagery to isolate regional vegetation dynamics for comparative analysis between the two decades. This approach mitigates soil reflectance interference, enhancing sensitivity to vegetation changes in sparsely vegetated environments.

## Results

### Floristic composition and conservation analysis

**Fig. 2:**
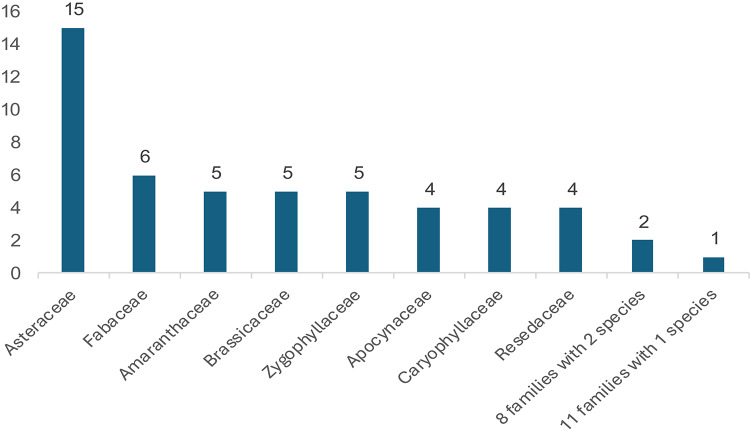
Graphical representation of plant families according to the numbers of the species collected from the studied area.

The floristic composition includes 75 species distributed across 27 plant families. Asteraceae (15 species) and Fabaceae (6 species) are the most species-rich. Amaranthaceae, Brassicaceae, and Zygophyllaceae each comprise five species; Apocynaceae, Caryophyllaceae, and Resedaceae each contain four. Eight families are represented by two species each, and 11 families are represented by a single species (Table 1; Fig. 2).

**Fig. 3:**
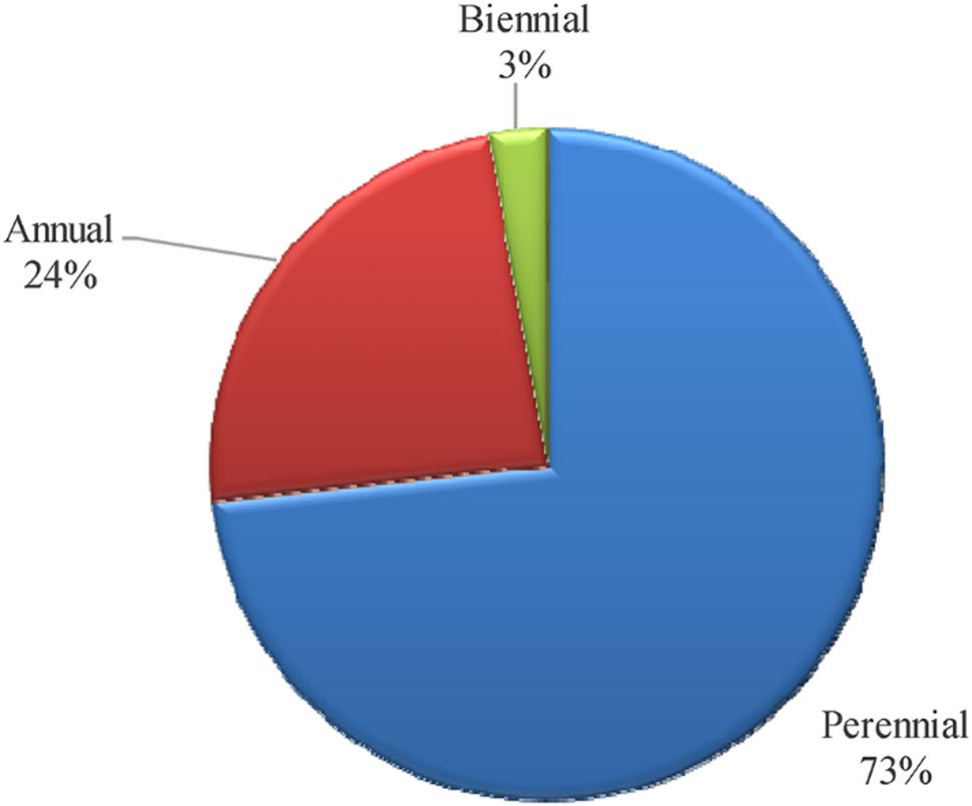
Life span of the recorded species.

**Fig. 4:**
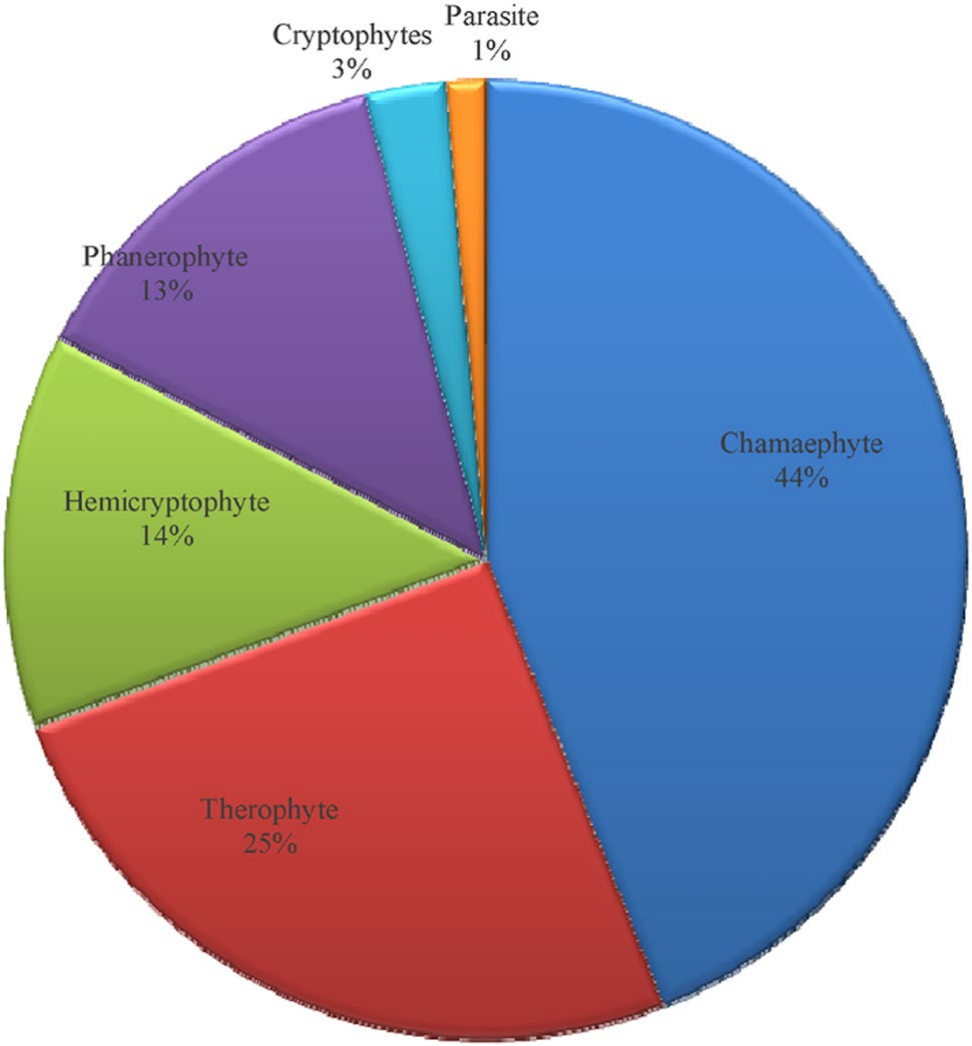
Life forms of the recoded species.

Perennial life spans dominate (55 species, 73%), while annuals (18 species, 24%) and biennials (2 species, 3%) are less common, indicating a flora structured to endure recurrent environmental stress (Table 1; Fig. 3). Life form analysis reveals a prevalence of chamaephytes (33 species, 44%) and therophytes (19 species, 25%), phanerophytes (10 species, 13%), hemicryptophytes (10 species, 13%), cryptophytes (2 species, 3%), and parasites (1 species, 1%). (Table 1; Fig. 4).

**Fig. 5:**
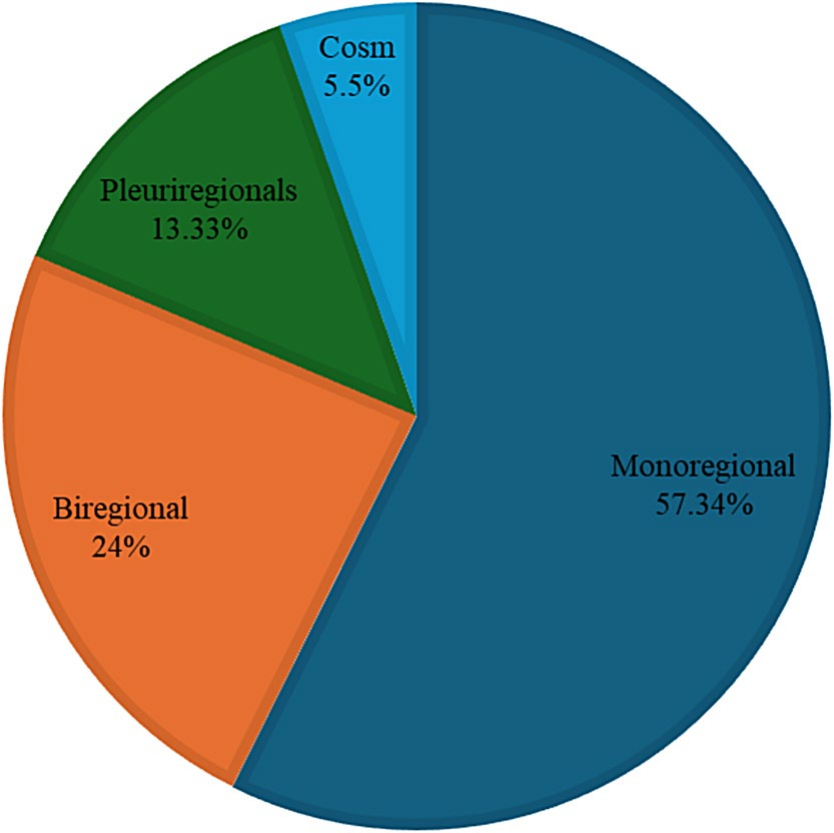
Floristic categories of the recorded species.

From a phytogeographical perspective, the documented species were categorized into four distributional groups: monoregional (57.34%), biregional (24%), pluriregional (13.33%), and cosmopolitan (5.3%), reflecting their respective geographic ranges. Phyto-geographical affinities highlight the Saharo–Arabian region (monoregional, biregional, pluriregional) as the primary contributor, with 53 species (71%). The results of the chorology of the recorded species are summarized in Tables 1 and 2; Fig. 5. Conservation status assessments reveal that 65 species (89%) remain unevaluated by the IUCN, while only 8 species (11%) are classified as Least Concern.

**Table 1:** List of recorded species in the study area during 2024.

Family	Species	Life form	Life span	Chorology	Conservation status according to IUCN
Amaranthaceae	*Ouret lanata* (L.) Kuntze	Th.	Pe.	IR-TR	N.E.
	*Haloxylon salicornicum* (Moq.) Bunge ex Boiss.	Ch.	Pe.	SA-AR	N.E.
	*Chenopodiastrum murale* (L.) S.Fuentes, Uotila & Borsch	Th.	An.	COSM	N.E.
	*Beta vulgaris* L.*	Th.	An.	ME + IR-TR + ER-SR	L.C.
	*Anabasis setifera* Moq.	Ch.	Pe.	SA-AR	N.E.
Apiaceae	*Deverra tortuosa* (Desf.) DC.	Ch.	Pe.	SA-AR	N.E.
	*Deverra triradiata* Hochst. ex Boiss.	Ch.	Pe.	SA-AR	N.E.
Apocynaceae	*Calotropis procera* (Aiton) W.T.Aiton	Ph.	Pe.	SA-AR + S-Z	L.C.
	*Cynanchum acutum* L.	He.	Pe.	ME + IR-TR	L.C.
	*Leptadenia pyrotechnica* (Forssk.) Decne.	Ph.	Pe.	SA-AR	L.C.
	*Pergularia tomentosa* L.	Ch.	Pe.	SA-AR	N.E.
Asteraceae	*Achillea fragrantissima* (Forssk.) Sch.Bip.	Ch.	Pe.	SA-AR + IR-TR	N.E.
	*Artemisia judaica* L.	Ch.	Pe.	SA-AR	N.E.
	*Asteriscus graveolens* (Forssk.) Less.	Ch.	Pe.	SA-AR	N.E.
	*Centaurea aegyptiaca* L.	Th.	An.	SA-AR	N.E.
	*Centaurea calcitrapa* L.*	Th.	An.	ME	N.E.
	*Echinops spinosissimus subsp. spinosissimus*	Ch.	Pe.	SA-AR + S-Z	N.E.
	*Iphiona mucronata* (Forssk.) Asch. & Schweinf.	Ch.	Pe.	SA-AR	N.E.
	*Launaea nudicaulis* (L.) Hook.f.	He.	Pe.	SA-AR	N.E.
	*Launaea spinosa* (Forssk.) Sch.Bip. ex Kuntze	Th.	Pe.	ME	N.E.
	*Pulicaria undulata* (L.) C.A.Mey.	Ch.	Pe.	SA-AR + S-Z	N.E.
	*Pulicaria undulata subsp. undulata*	Ch.	Pe.	SA-AR	N.E.
	*Reichardia tingitana* (L.) Roth	Th.	An.	ME + IR-TR + SA-AR	N.E.
	*Senecio glaucus subsp. coronopifolius* (Maire) C.Alexander	Th.	An.	ME + IR-TR + ER-SR	N.E.
	*Senecio vulgaris* L.**	Th.	An.	ME + IR-TR + ER-SR	N.E.
	*Sonchus oleraceus* L.**	Th.	An.	COSM	N.E.
Boraginaceae	*Heliotropium arbainense* Fresen.	Ch.	Pe.	SA-AR	N.E.
	*Trichodesma africanum* (L.) Sm.	Ch.	Pe.	SA-AR + S-Z	N.E.
Brassicaceae	*Diplotaxis harra* (Forssk.) Boiss.	Th.	An.	SA-AR + IR-TR	L.C.
	*Farsetia aegyptia* Turra	Ch.	Pe.	SA-AR	N.E.
	*Matthiola arabica* Boiss.	He.	Pe.	SA-AR	N.E.
	*Sisymbrium irio* L.	Th.	An.	ME + IR-TR + ER-SR	N.E.
	*Zilla spinosa* (L.) Prantl	Ch.	Pe.	SA-AR	N.E.
Capparaceae	*Capparis spinosa* L.	Ph.	Pe.	ME + IR-TR	L.C.
Caryophyllaceae	*Gymnocarpos decandrus* Forssk.	Ch.	Pe.	ME + SA-AR + IR-TR	N.E.
	*Gypsophila capillaris* (Forssk.) C.Chr.	Ch.	Pe.	IR-TR	N.E.
	*Paronychia argentea* Lam.	He.	An.	ME	N.E.
	*Spergularia marina* (L.) Besser	Th.	Bi.	ME + IR-TR + ER-SR	L.C.
Cleomaceae	*Cleome arabica* L.	Ch.	Pe.	S-Z	N.E.
Cleomaceae	*Cleome droserifolia* (Forssk.) Delile.	Ch.	Pe.	SA-AR + IR-TR	N.E.
Convolvulaceae	*Convolvulus hystrix* Vahl	Ch.	Pe.	SA-AR + S-Z	N.E.
Ephedraceae	*Ephedra alata* Decne.	Ph.	Pe.	ME + SA-AR	L.C.
Euphorbiaceae	*Chrozophora oblongifolia* (Delile) A.Juss. ex Spreng.	Ch.	Pe.	S-Z	N.E.
	*Euphorbia exigua subsp. exigua*	Th.	An.	SA-AR	N.E.
Fabaceae	*Alhagi maurorum* Medik.	Ch.	Pe.	SA-AR	N.E.
	*Crotalaria aegyptiaca* Benth.	Ch.	Pe.	S-Z	N.E.
	*Lotus corniculatus* L.**	He.	Bi.	ME + IR-TR + ER-SR	N.E.
	*Lotus hebricus* Hochst. ex Brand	He.	An.	SA-AR	N.E.
	*Retama raetam* (Forssk.) Webb & Berthel.	Ph.	Pe.	SA-AR	N.E.
	*Trigonella stellata* Forssk.	Th.	An.	ME + SA-AR + IR-TR	N.E.
Geraniaceae	*Erodium salzmannii subsp. salzmannii*	He.	Pe.	SA-AR	N.E.
Lamiacea	*Lavandula coronopifolia* Poir.	Ch.	Pe.	SA-AR	N.E.
	*Salvia spinosa subsp. spinosa*	Ch.	Pe.	SA-AR + IR-TR	N.E.
Mimosaceae	*Vachellia tortilis subsp. raddiana* (Savi) Kyal.	Ph.	Pe.	SA-AR + S-Z	N.E.
Nitrariaceae	*Peganum harmala* L.	Ch.	Pe.	SA-AR + IR-TR	N.E.
Orobanchaceae	*Orobanche cernua* Loefl.	Pa.	Pe.	ME + IR-TR	N.E.
Plantaginaceae	*Kickxia aegyptiaca* (L.) Nábělek	Ch.	Pe.	SA-AR	N.E.
Poaceae	*Panicum turgidum* Forssk.	Cr.	Pe.	SA-AR	N.E.
	*Phragmites australis* (Cav.) Trin. ex Steud.**	Cr.	Pe.	COSM	L.C.
Polygonaceae	*Calligonum aphyllum* (Pall.) Gürke	Ph.	Pe.	SA-AR	N.E.
	*Rumex vesicarius* L.	Th.	An.	ME + SA-AR + S-Z	N.E.
Primulaceae	*Lysimachia arvensis* (L.) U.Manns & Anderb.	Th.	An.	COSM	N.E.
Resedaceae	*Caylusea hexagyna* (Forssk.) M.L.Green	Th.	An.	SA-AR	N.E.
	*Ochradenus baccatus* Delile	Ph.	Pe.	SA-AR	L.C.
	*Reseda muricata* C.Presl	Ch.	Pe.	SA-AR	N.E.
	*Reseda pruinosa* Delile	Th.	An.	SA-AR	N.E.
Solanaceae	*Hyoscyamus muticus* L.	Ch.	Pe.	SA-AR + IR-TR	N.E.
	*Lycium shawii* Roem. & Schult.	Ph.	Pe.	SA-AR + S-Z	L.C.
Tamaricaceae	*Tamarix nilotica* (Ehrenb.) Bunge	Ph.	Pe.	SA-AR	L.C.
Urticaceae	*Forsskaolea tenacissima* L.	Ch.	Pe.	SA-AR + S-Z	N.E.
Zygophyllaceae	*Zygophyllum arabicum* (L.) Christenh. & Byng	He.	Pe.	SA-AR	N.E.
	*Zygophyllum coccineum* L.	Ch.	Pe.	SA-AR	N.E.
	*Zygophyllum decumbens* Delile	Ch.	Pe.	SA-AR	N.E.
	*Zygophyllum molle* (Delile) Christenh. & Byng	He.	Pe.	SA-AR	N.E.
	*Zygophyllum scabrum* (Forssk.) Christenh. & Byng	He.	Pe.	SA-AR	N.E.

Species indicated by their families, Life forms: Therophyte = Th, Hemicryptophyte = He, Chamaephyte = Ch, Phanerophyte = Ph, Cryptophytes = Cr, and Parasite = Pa. Phytogeographical affinity; Cosmopolitan = COSM, Euro - Siberian = ER-SR, Irano - Turanian = IR-TR, Mediterranean = ME, Saharo–Arabian = SA-AR, and Sudano–Zambesian = S-Z. Conservation status according to IUCN: Not evaluated = N.E., and Least Concern = L.C.

*Alien species.

**Invasive species.

**Table 2:** Number of species belonging to the main floristic categories and their percentages.

Floristic category	No. of species (%)
Cosm	4 (5.33)
Monoregional	43 (57.34)
SA-AR	35 (46.67)
ME	3 (4)
S-Z	3 (4)
IR-TR	2 (2.67)
Biregional	42 (42)
SA-SR + S-Z	8 (10.67)
SA-SR + IR-TR	6 (8)
ME + IR-TR	3 (4)
ME + SA-SR	1 (1.33)
Pleuriregional	10 (13.33)
ME + IR-TR + ER-SR	7 (9.33)
ME + SA-AR + IR-TR	2 (2.67)
ME + SA-AR + S-Z	1 (1.33)

### Soil analyses

**Fig. 6:**
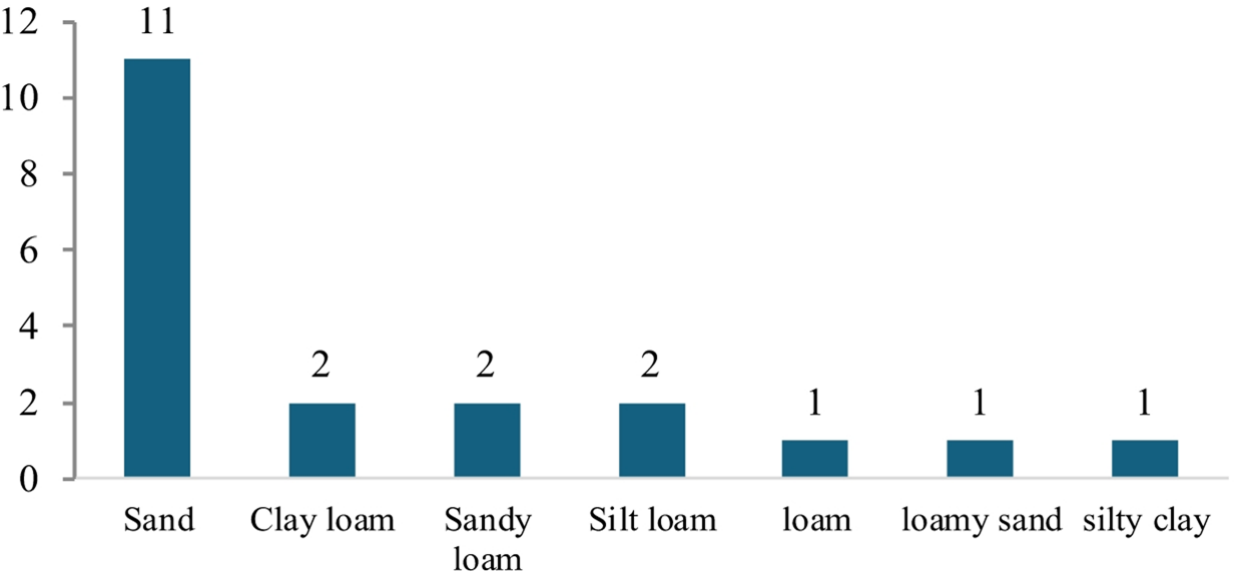
Soil texture spectrum of the selected stands in the study area.

The results of descriptive statistics for soil samples collected from the studied stands are summarized in Table 3. The soil analysis results across the 20 stands reveal significant variability in physicochemical properties, reflecting diverse soil conditions. The results of Soil texture classifications are determined by particle size distribution. Sand content ranged from 4.5% (Stand 9) to 98.2% (Stand 11), clay from 1.2% (Stand 7) to 43% (Stand 9), and silt from 0.3% (Stand 19) to 67.5% (Stand 8). Soil mechanical analysis showed that soil texture may be Sand, clay loam, sandy loam, silty loam, loam, loamy sand, or silty clay. Sandy soil was represented by 55% of the studied stands (11 stands), clay loam, sandy loam, and silty loam were represented by 10% (2 stands) each, while loam, loamy sand, and silty clay were represented by 5% (1 stand) each (Fig. 6).

Soil saturation percentage (SP) showed that the minimum SP was 20% (stand 12), while the maximum was 50% (stand 9), with an average of 35.35% indicating variations in water holding capacity among stands. The pH values ranged from slightly acidic to moderately alkaline (7.23–8.60), with most soils falling within the neutral to alkaline range, indicative of calcareous or sodic influences.

Electrical conductivity (EC) varied markedly, from 0.71 dS/m (Stand 19) to 131.20 dS/m (Stand 2), with an average of 10.79 dS/m, highlighting extremes in salinity. Stand 2 exhibited severe salinity (EC > 8 dS/m. consistent with its exceptionally high total dissolved salts (TDS: 83968 ppm) and elevated sodium (852.17 meq/L), chloride (1101.7meq/L), and sulfate (319.64 meq/L) concentrations. In contrast, non-saline soil (EC < 2 dS/m) was observed in Stands 12, 19, and 20, with TDS values below 1,500 ppm.

The chemical composition of the soil extracts from the 20 investigated stands showed considerable variation in both cationic and anionic constituents (Table 3). Among the cations, sodium (Na⁺) exhibited the highest average concentration (49.92 meq L⁻¹), with values ranging from 0.82 to 852.17 meq L⁻¹, followed by calcium (Ca²⁺) with an average of 43.01 meq L⁻¹ (range: 2.98–333.30 meq L⁻¹). Magnesium (Mg²⁺) recorded a mean value of 22.45 meq L⁻¹, fluctuating between 3.75 and 243.59 meq L⁻¹. Potassium (K⁺) was generally low, averaging 2.35 meq L⁻¹, with a minimum of 0.25 and a maximum of 25.26 meq L⁻¹.

Regarding the anions, chloride (Cl⁻) was the most dominant, with an average concentration of 80.30 meq L⁻¹ and a wide range extending from 3.39 to 1101.70 meq L⁻¹. Sulfate (SO₄²⁻) followed, with a mean of 31.91 meq L⁻¹ and values between 2.06 and 319.64 meq L⁻¹. Bicarbonate (HCO₃⁻) showed relatively low levels, averaging 5.52 meq L⁻¹ (range: 1.89–33.02 meq L).

Overall, the results indicate that Na⁺ and Cl⁻ were the predominant ions in the studied soils, reflecting a clear trend towards salinity, whereas K⁺ and HCO₃⁻ contributed the least to the ionic composition. The wide ranges observed for several ions, particularly Na⁺ and Cl⁻, highlight spatial variability in soil salinity across the studied stand. The total dissolved salts (TDS) ranged between 456.32 ppm as a minimum value in Stand 19, a maximum value of 83,968 ppm in Stand 2, with an average of 6903.936 ppm while the Organic matter content was generally low (0.07–1.31%), with the highest value in Stand 9 (1.31%) and the lowest value in Stand 0.07%.

**Table 3:** A descriptive statistical summary of soil analysis data of 20 samples from the surveyed stands.

Soil parameter	Range (minimum–maximum)	Mean ± standard deviation	Median
Sand (%)	93.65 (4.5-98.15)	71.311 ± 32.874	91.65
Clay (%)	42.167 (1.156–43.323)	11.777 ± 11.741	5.719
Silt (%)	67.203 (0.288–67.491)	16.913 ± 22.46	4.694
Soil saturation percentage	35 (20–55)	35.35 ± 10.122	30
pH	1.37 (7.23–8.6)	8.067 ± 0.458	8.155
Electrical conductivity (dS/m)	130.487 (0.713–131.2)	10.787 ± 28.489	3.818
Calcium (m eq/L)	330.357 (2.976-333.333)	43.006 ± 71.131	23.81
Magnesium (m eq/L)	239.835 (3.755–243.59)	22.451 ± 52.357	9.455
Sodium (m eq/L)	851.353 (0.82-852.174)	49.917 ± 188.958	5.652
Potassium (m eq/L)	25.005 (0.253–25.258)	2.35 ± 5.526	0.866
Bicarbonates (m eq/L)	31.132 (1.887–33.019)	5.519 ± 6.853	3.538
Chlorides (m eq/L)	1098.305 (3.39-1101.695)	80.297 ± 241.509	18.644
Sulfates (m eq/L)	317.585 (2.056-319.641)	31.908 ± 68.219	18.964
Sodium absorption ratio	49.727 (0.447–50.175)	4.017 ± 10.907	1.406
Total dissolved salts (ppm)	83511.68 (456.32–83968)	6903.936 ± 18232.656	2443.52
Organic matter (%)	1.24 (0.07–1.31)	0.447 ± 0.283	0.42

### Vegetation: soil relationships

**Fig. 7:**
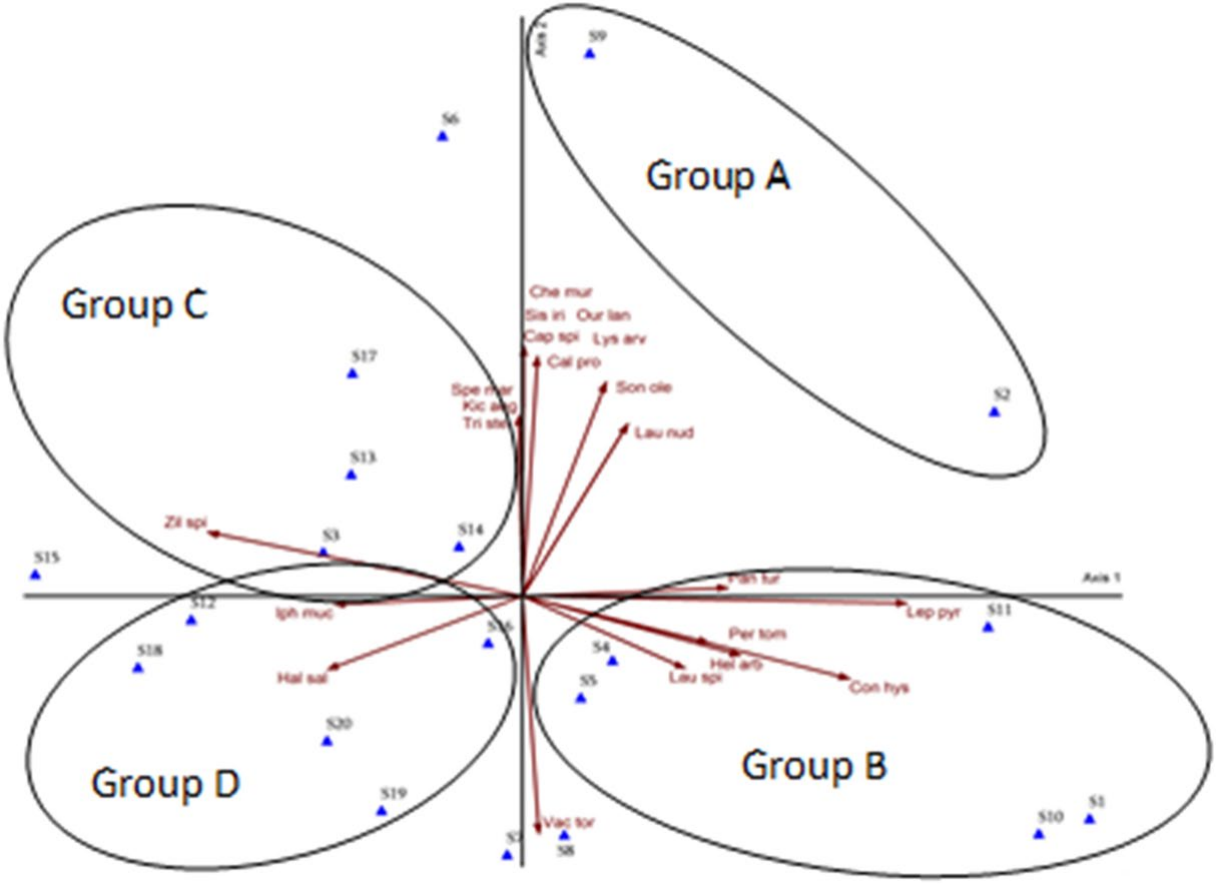
DCA ordination diagram for the 20 studied stands, correlation with different plant indicators. Cal pro = Calotropis procera, Cap spi = Capparis spinosa, Che mur = Chenopodiastrum murale, Con hys = Convolvulus hystrix, Hal sal = Haloxylon salicornicum, Hel arb = Heliotropium arbainense, Iph muc = Iphiona mucronate, Kic aeg = Kickxia aegyptiaca, Lau nud = Launaea nudicaulis, Lau spi = Launaea spinosa, Lep pyr = Leptadenia pyrotechnica, Lys arv = Lysimachia arvensis, Our lan = Ouret lanata, Pan tur = Panicum turgidum, Per tom = Pergularia tomentosa, Sis iri = Sisymbrium irio, Son ole = Sonchus oleraceus, Spe mar = Spergularia marina, Tri ste = Trigonella stellata, Vac tor = Vachellia tortilis subsp. raddiana, Zil spi = Zilla spinose.

To assess the correlations between plant indicator species and vegetation stands, as well as the influence of edaphic factors on stand composition, a multivariate Detrended Correspondence Analysis (DCA) was performed. This analysis aimed to identify gradients in vegetation structure linked to species distribution and to evaluate the relative contribution of specific soil parameters in differentiating stand groups. The results were used to determine the dominant edaphic drivers shaping ecological variation within each stand cluster. The Detrended Correspondence Analysis (DCA) revealed distinct plant indicator species correlations across the studied stand groups. Group A consists of stands 2 and 9 exhibited strong correlations with *Chenopodiastrum murale*, *Sisymbrium irio*, *Ouret lanata*, *Capparis spinosa*, *Lysimachia arvensis*, *Calotropis procera*, *Sonchus oleraceus*, *Launaea nudicaulis*, and *Panicum turgidum*. Group B consists of stands 1, 4, 5, 8, 10, and 11 were primarily correlated to *Leptadenia pyrotechnica*, *Pergularia tomentosa*, *Convolvulus hystrix*, *Heliotropium arbainense*, *Launaea spinosa*, and *Vachellia tortilis* subsp. *raddiana*. Group C consists of stands 3, 6, 13, 14, 15, and 17 showed strong associations with *Spergularia marina*, *Kickxia aegyptiaca*, *Trigonella stellata*, and *Zilla spinosa*, while Group D consists of stands 7, 12, 16, 18, 19, and 20 were characterized by the presence of *Haloxylon salicornicum* and *Iphiona mucronata*. These correlations highlight species-specific affinities to particular stand clusters, reflecting underlying ecological gradients and edaphic influences (Fig. 7).

**Fig. 8:**
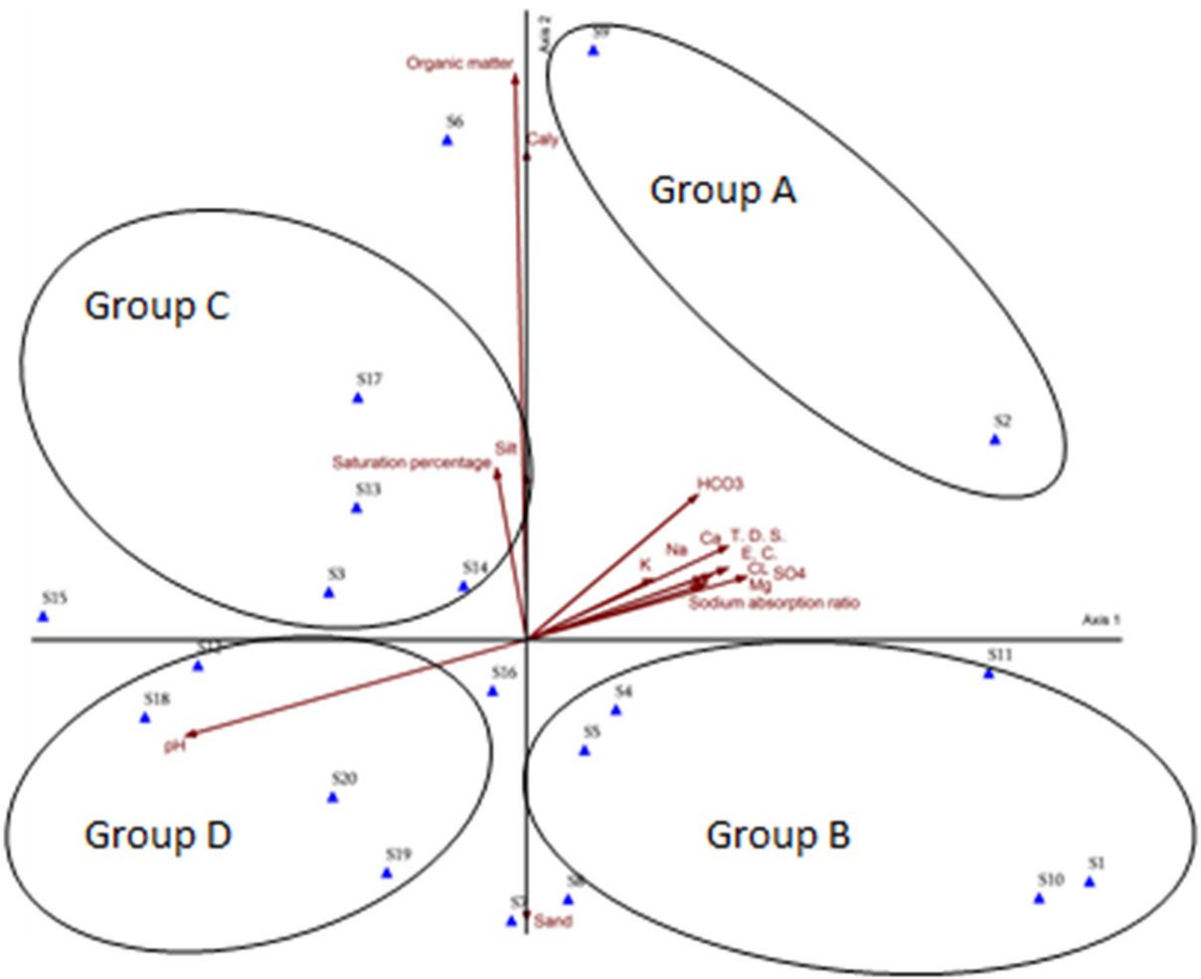
DCA ordination diagram for the 20 studied stands, correlation with different soil factors.

The analysis identified distinct edaphic drivers across the stand groups. Group A (Stands 2 and 9) was most strongly influenced by salinity-related parameters, including electrical conductivity, total dissolved salts, and Sodium Absorption Ratio (SAR), as well as elevated concentrations of calcium, magnesium, sodium, potassium, chloride, sulfate, and bicarbonate ions. Group C (Stands 3, 6, 13, 14, 15, and 17) exhibited significant correlations with organic matter content and soil saturation percentage, reflecting conditions linked to moisture retention and nutrient dynamics. In contrast, group D (stands 7, 12, 16, 18, 19, and 20) was primarily associated with soil pH, highlighting the role of acidity-alkalinity gradients in shaping their edaphic profiles. These results underscore the differential influence of soil physicochemical properties in structuring stand-specific environmental conditions (Fig. 8).

### Vegetation change

**Fig. 9:**
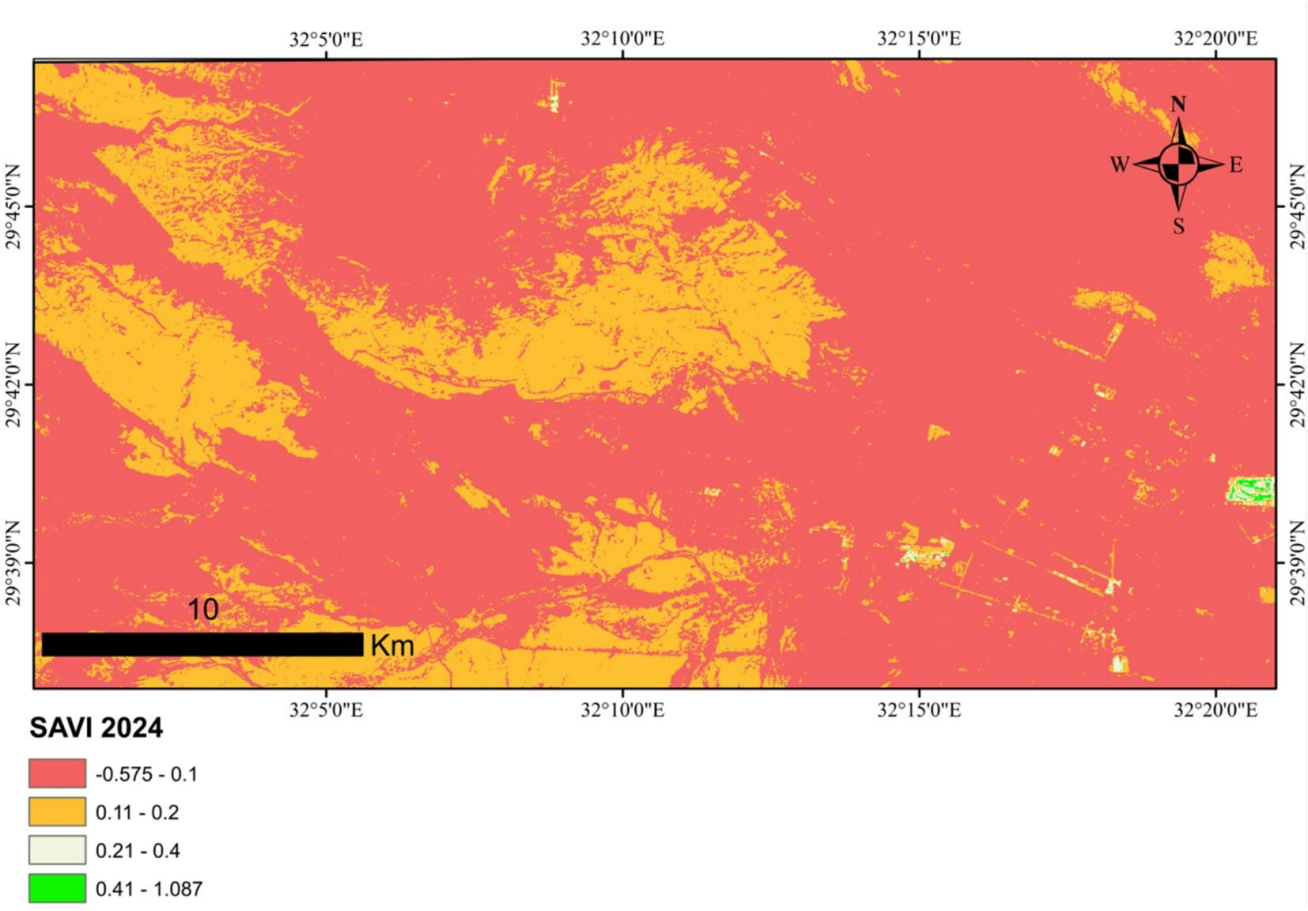
SAVI maps for 2024 (the map was created using ArcGIS 10.5 program).

**Fig. 10:**
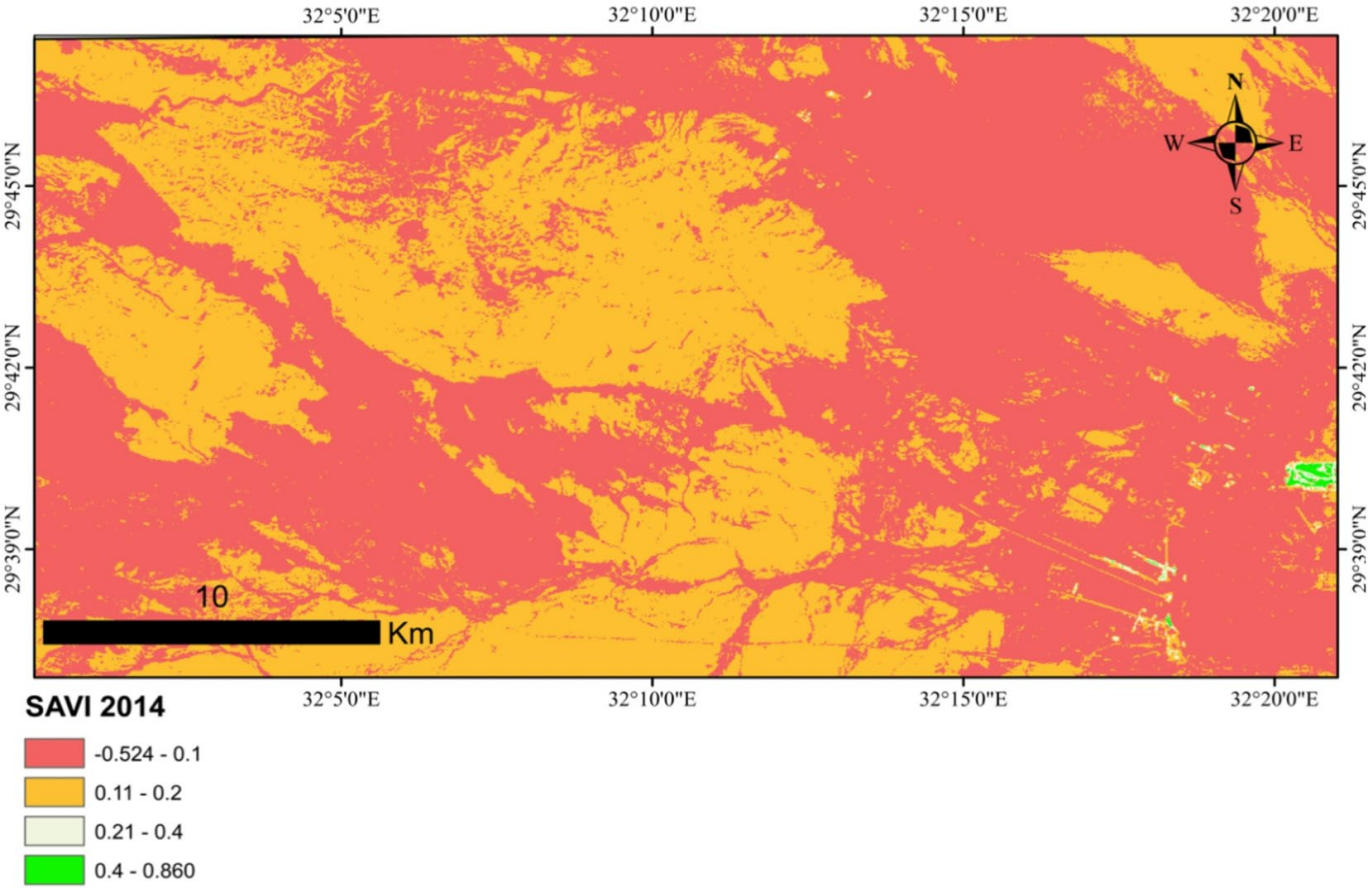
SAVI maps for 2014 (the map was created using ArcGIS 10.5 program).

Soil Adjusted Vegetation Index (SAVI) for 2014 ranged from − 0.523911 to 0.860437, and for 2024, it ranged from − 0.574714 to 1.08698 (Figs. 9 and 10). The observed increase in the SAVI values between 2014 and 2024 may be attributed to several factors. One potential explanation is the occurrence of natural vegetation recovery within the area. Alternatively, the proliferation of invasive plant species, which can colonize and dominate sections of the landscape, may also contribute to an elevated vegetation index.

## Discussion

The dominance of Asteraceae and Fabaceae aligns with their adaptability to arid environments, as both families include species with traits such as drought tolerance. The observed results align with floristic patterns reported in adjacent regions of the Eastern Desert of Egypt. In Wadi Hagul, Bedair et al.^19^ identified Asteraceae and Brassicaceae as the most frequent plant families, while Marie^40^ documented Asteraceae and Zygophyllaceae as dominant. Similarly, Abdelaal^18^ reported Asteraceae and Poaceae as the predominant families in the same area. Asteraceae’s prevalence extends beyond the study area, with Salama et al.^17^ highlighting its dominance in other Eastern Desert wadis, such as Wadi Asyouti and Wadi Habib. The Asteraceae family was most represented in Wadi Degla and on the Cairo-Suez road^41^. This variability in secondary dominant families may reflect ecological gradients or methodological differences across studies. Asteraceae’s ecological success in arid environments is attributed to its abundance of salt-tolerant and xerophytic species^42^. Asteraceae constitutes a major component of Egypt’s flora, encompassing 98 genera and 234 species^43^, a taxonomic richness that likely underpins its widespread distribution and adaptability in desert ecosystems.

Perennial species constituted the dominant plant life span (73%) in the current study, a pattern consistent with vegetation surveys conducted in analogous hyperarid regions of Egypt’s Eastern Desert^18,19,40,44^. In contrast, annual species exhibited notably low representation along the old Katameya-Ain Sokhna Road, likely attributable to reduced precipitation during the study period. Such interannual variability in rainfall—common in arid ecosystems—can disproportionately affect short-lived annuals, which rely on timely moisture availability for germination and establishment.

The life forms of plant species are primarily shaped by their adaptation to environmental conditions, particularly climate^45^. In desert ecosystems, plant life forms exhibit a strong association with precipitation patterns^46,47^ and demonstrate correlations with landform characteristics and topography^48,49^. The predominance of chamaephytes (44%) and therophytes (25%) is consistent with broader ecological patterns observed in arid and semi-arid regions, as documented in studies from Egypt^19,50^, Iran^51^, and Saudi Arabia^52^. This life-form distribution reflects adaptive strategies to water scarcity and environmental stressors characteristic of such ecosystems.

This distribution underscores the study area’s position where Saharo–Arabian species dominate (65.8%) but interact with neighboring floristic regions. Saharo-Arabian species dominated the floristic composition, reflecting the study area’s biogeographical affinity with hyperarid regions of North Africa, the Arabian Peninsula. This pattern aligns with vegetation surveys in analogous Eastern Desert ecosystems^18,19,40,44^. As well as these results are also consistent with numerous studies conducted in coastal wadis of South Sinai, Egypt^53^. The prevalence of Saharo-Arabian taxa underscores their role as ecological indicators of desert conditions, as their morphological and physiological traits are specialized to withstand extreme aridity, temperature fluctuations, and soil salinity^54^.

Limited IUCN Red List assessments for documented species underscore critical gaps in conservation prioritization, particularly for endemic^14^. Future research should integrate long-term environmental monitoring and mapping of anthropogenic disturbances to develop evidence-based management frameworks to conserve and protect plant species from harm. Reevaluating plant species distributions and population dynamics, particularly in arid ecosystems, is critically important to inform effective conservation strategies for safeguarding native and endemic flora.

The study documented two alien plant species [*Beta vulgaris*, *Centaurea calcitrapa*; -^31^ and four invasive species [*Senecio vulgaris*,* Sonchus oleraceus*,* Lotus corniculatus*, and *Phragmites australis*]^32^. Their spread disrupts ecosystems by outcompeting native flora for resources (light, water, nutrients) and suppressing regeneration via rapid growth, high reproduction, and allelopathy. This reduces biodiversity, alters habitats, diminishes food/habitat availability for fauna, and degrades resilience through soil chemistry and fire regime shifts. Recent studies underscore that these impacts exacerbate local species declines and increase extinction risks, particularly in biodiversity hotspots^55^. Elevated soil moisture, salinity, and temperature linked to climate change can create favorable abiotic conditions for invasive species establishment, potentially enabling their competitive displacement of native plant communities^56^.

The interplay of salinity, organic matter, and pH as edaphic drivers aligns with global patterns of soil-plant interactions: salinity and sodicity shape vegetation zonation in arid systems^57^. The soil analysis along the Katameya-Ain Sokhna road highlights geochemical and textural similarities to Egypt’s Eastern Desert, driven by analogous climatic and geological conditions. The pH range (7.23–8.60) aligns with slightly alkaline to moderately alkaline soils in Wadi Hagul^19^, reflecting calcareous parent materials and limited leaching in hyper-arid environments. Such alkalinity, typical of Egyptian desert soils, arises from carbonate dissolution and sodium accumulation^58^. The high soil salinity in many stands, such as stand 2 (EC: 131.20 dS/m) near the Red Sea in Egypt, is primarily attributed to arid climatic conditions, seawater intrusion, and evaporite deposition. In this hyper-arid region, high evaporation rates concentrate salts in the soil as groundwater, and sometimes seawater (due to tidal surges or subsurface seepage), evaporates, leaving behind dissolved minerals. In addition, the tectonic history of the Red Sea has left ancient marine evaporites (salt deposits) subsurface, which dissolve and travel upward by capillary action. Low rainfall and meager freshwater inflow also prevent salt leaching, resulting in persistently saline soils, particularly in coastal sabkhas (salt flats) and depressions. The results of soil texture analysis showed that the sand content was the highest in most of the studied sites, which is largely consistent with Bedair et al.^19^, who found that the percentage of sand in all soil samples in the studied sites in Wadi Hagul exceeded 81%.

Detrended Correspondence Analysis (DCA) is a widely applied ordination technique for distinguishing plant communities through the quantification of species compositional variation along ecological gradients or environmental variables [59, 60. 61]. The DCA revealed plant species distribution across vegetation stands aligned with key edaphic gradients. Group A (stands 2 and 9), dominated by halophytes correlated with elevated salinity (electrical conductivity, sodium ions) and sodicity (SAR), reflects adaptations like ion sequestration^62^. High SAR disrupts soil structure, limiting water availability for non-tolerant species^63^. Group B (stands 1, 4, 5, 8, 10, and 11), characterized by the xerophytes *Leptadenia pyrotechnica* and *Vachellia tortilis* subsp. *raddiana*, reflects adaptation to aridity and low organic matter, with deep-rooting and nitrogen-fixing traits aiding survival in nutrient-poor soils. Group C (stands 3, 6, 13, 14, 15, and 17), linked to *Spergularia marina* and *Kickxia aegyptiaca*, correlated with organic matter and soil saturation, as organic carbon enhances moisture retention. Group D (stands 7, 12, 16, 18, 19, and 20), dominated by *Haloxylon salicornicum* and *Iphiona mucronata*, associated with alkaline pH gradients, reflects adaptations to calcareous soils where high pH limits nutrient bioavailability but selects for specialized uptake mechanisms.

The increase in plant cover (SAVI) during the study year (2024), compared to the previous year (2014), can be attributed to a number of factors. These environmental factors include the potential for the growth of some invasive species.

Egypt’s wild flora harbors diverse species with documented pharmacological properties and socioeconomic relevance, including bioactive compounds that may offer therapeutic potential for addressing various human ailments. These botanical resources represent underutilized reservoirs of phytochemical diversity, which could inform drug discovery pathways while supporting sustainable resource development^64–69^. The study area was found to contain 16 plant species, all of which exhibit medicinal properties with documented therapeutic applications. This observation underscores the ecological and ethnobotanical significance of the region’s flora (Table 4).

**Table 4:** Plant species recorded in the study area and their medicinal uses.

Medicinal species	Uses	References
*Halexylon salicornicum*	Grazing and antimicrobial activity	^70^
*Anabasis setifera*	Antimicrobial, anticancer, antioxidant activities	^71^
*Deverra tortuosa*	Antioxidant, antimicrobial activities	^72^
*Deverra triradiata*	Antioxidant, anti-inflammatory activities	^73^
*Calotropis procera*	Antioxidant, anti-inflammatory, antitumoral, hypoglycemic, gastric protective, antimicrobial, insecticide, antifungal, anti-parasitic activities	^74^
*Cynanchum acutum*	Antibacterial, antioxidant activities	^75^
*Leptadenia pyrotechnica*	Antioxidant activity	^76^
*Pergularia tomentosa*	Antioxidant, anti-inflammatory activities	^77^
*Achillea fragrantissima*	Antibacterial, anti-inflammatory activities	^78^
*Artemisia judaica*	Antioxidant, antispasmodic activities	^79^
*Asteriscus graveolens*	Antifungal activity	^80^
*Launaea nudicaulis*	Antibacterial, antibiofilm, anticancer activities	^81^
*Capparis spinosa*	Anti-inflammatory, antioxidant activities	^82^
*Tamarix nilotica*	anti-cancer activity	^83^
*Lycium shawii*	Anti-malarial activity	^84^
*Rumex vesicarius*	Antibiofilm, antibacterial, antioxidant activities	^85^
*Spergularia marina*	Antioxidants, antimicrobials, anticancer activities	^86^
*Sisymbrium irio*	Antifungal, antibacterial activities	^87^

## Conclusions

This study was conducted in the old Katameya-Ain Sokhna Road area, an unprotected region within Egypt’s Eastern Desert. It aimed to evaluate vegetation cover, analyze relationships between indicator plant species across study stands, and assess soil factors influencing stand groupings. The increase in vegetation cover in the study year compared to previous years may be due to many factors, such as the increased spread of invasive plants. Sixteen plant species were identified within the study area, each possessing documented medicinal properties and therapeutic applications.

## Supplementary Information

Below is the link to the electronic supplementary material.

Supplementary Material 1

## Data Availability

Data from the present article are available in the paper.
