# Supplementary material for: Floristic assessment and soil-vegetation dynamics in an arid zone: a case study of the old Katameya-Ain Sokhna Road, Eastern Desert, Egypt
Source: Sci Rep. 2025 Oct 29;15:37742. doi: 10.1038/s41598-025-22507-z (PMC12572113; doi:10.1038/s41598-025-22507-z)
Supplement: Supplementary file 1 — Supplementary Material 1 [file 41598_2025_22507_MOESM1_ESM.pdf]

Materials and Methods

1. Climate Data of the study area

Climate data for the past 30 years were extracted from **www.meteoblue.com**. They include mean maximum temperature (°C), mean minimum temperature (°C), and precipitation (mm) ( **Figure 1**). Wind direction and velocity (km/h) are provided in **Figure 2**.

A map of the study area will be added showing the elevation above sea level.

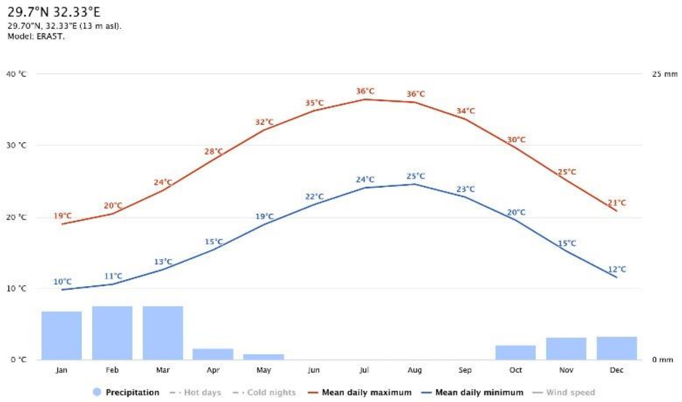


Fig. 1. Klima diagram showing average annual temperature range and rainfall of the study area.

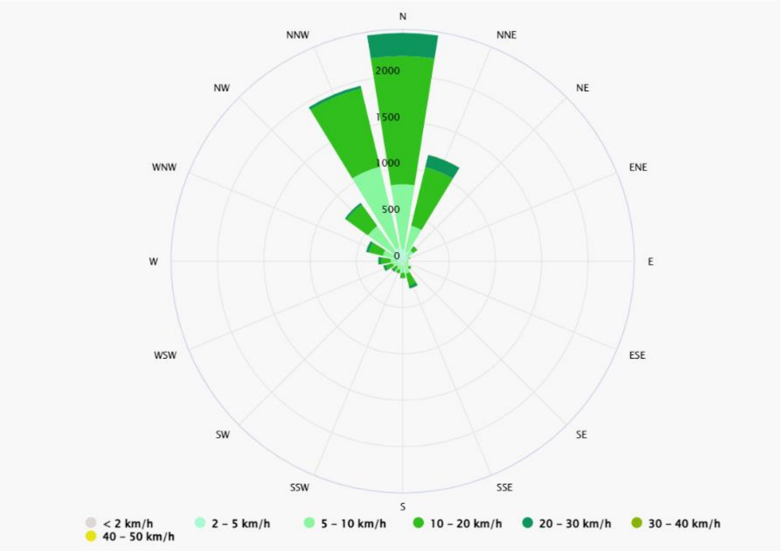


Fig. 2. Wind rose showing wind direction of the study area.

2. Floristic Composition

Table 1. Global Positioning System (GPS) coordinates of the selected 20 stands within the study area

| Stand | Latitude (N) | Longitude (E) | Elevation above sea level (meters) |
|---|---|---|---|
| 1 | 29° 41' 48.01" | 32° 20' 2.00" | 13 |
| 2 | 29° 42' 47.99" | 32° 17' 35.02" | 39 |
| 3 | 29° 44' 43.01" | 32° 14' 10.00" | 85 |
| 4 | 29° 46' 9.01" | 32° 11' 58.00" | 112 |
| 5 | 29° 46' 22.01" | 32° 10' 36.98" | 124 |
| 6 | 29° 46' 22.01" | 32° 2' 52.01" | 214 |
| 7 | 29° 46' 12.00" | 32° 1' 29.00" | 226 |
| 8 | 29° 39' 6.01" | 32° 16' 8.00" | 48 |
| 9 | 29° 37' 58.01" | 32° 18' 19.01" | 23 |
| 10 | 29° 42' 33.01" | 32° 19' 14.02" | 24 |
| 11 | 29° 42' 33.01" | 32° 15' 49.00" | 51 |
| 12 | 29° 45' 2.02" | 32° 13' 40.01" | 91 |
| 13 | 29° 46' 9.01" | 32° 11' 56.00" | 112 |
| 14 | 29° 46' 19.98" | 32° 10' 49.01" | 124 |
| 15 | 29° 46' 18.98" | 32° 10' 30.00" | 127 |
| 16 | 29° 46' 23.99" | 32° 2' 53.02" | 214 |
| 17 | 29° 46' 19.99" | 32° 1' 56.00" | 232 |
| 18 | 29° 46' 12.00" | 32° 1' 30.00" | 226 |
| 19 | 29° 46' 13.01" | 32° 1' 26.00" | 230 |
| 20 | 29° 46' 9.01" | 32° 0' 27.00" | 241 |

2. Select quadrats on stands

According to Braun – Blanquet (1964), Mueller-Dombois and Ellenberg (1974) and Shukla, and Chandel, (1989) in each stand 4 quadrates (10 × 10 m) were setup to determine the vegetation parameters and IVI

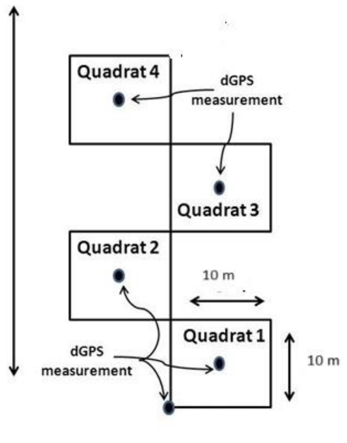


**Figure 3** A schematic illustration for the vegetation survey method.

Braun-Blanquet, J. (1964). Plant Sociology. Translated by G.D. Fuller and H.S. Connard, McGraw-Hill Book Co., Inc. New York, London. 439 pp.

Mueller-Dombois, D. and Ellenberg, H. (1974). Aims and methods of vegetation ecology. John Wiley & Sons, New York, 547 pp.

Shukla, K. S. and Chandel, P. S. (1989). Plant ecology and soil science. S. Chand and Co. New Delhi, India, 552 pp.

# Results

## Vegetation – soil relationships

Table 2. Groups resulting from DCA analysis and the most common plant Indicators for each group of stands

| Group | Indicator species | Family |
|---|---|---|
| A | *Chenopodiastrum murale* | Amaranthaceae |
| | *Ouret lanata* | |
| | *Calotropis procera* | Apocynaceae |
| | *Sonchus oleraceus* | Asteraceae |
| | *Launaea nudicaulis* | |
| | *Sisymbrium irio* | Brassicaceae |
| | *Capparis spinosa* | Capparaceae |
| | *Panicum turgidum* | Poaceae |
| | *Lysimachia arvensis* | Primulaceae |
| B | *Leptadenia pyrotechnica* | Apocynaceae |
| | *Pergularia tomentosa* | |
| | *Convolvulus hystrix* | Convolvulaceae |
| | *Heliotropium arbainense* | Boraginaceae |
| | *Launaea spinosa* | Asteraceae |
| | *Vachellia tortilis* subsp. *raddiana* | Fabaceae |
| C | *Spergularia marina* | Caryophyllaceae |
| | *Kickxia aegyptiaca* | Plantaginaceae |
| | *Trigonella stellata* | Fabaceae |
| | *Zilla spinosa* | Brassicaceae |
| D | *Iphiona mucronate* | Asteraceae |
| | *Haloxylon salicornicum* | Amaranthaceae |

Table 3. Groups resulting from DCA analysis and the most common soil parameter for each group of stands

| Group | Soil Parameter |
|---|---|
| A | Clay (%) |
| | Bicarbonates (m eq / L) |
| | Potassium (m eq / L) |
| | Sodium (m eq / L) |
| | Calcium (m eq / L) |
| | Total dissolved salts (ppm) |
| | Electrical conductivity (dS/m) |
| | Chlorides (m eq / L) |
| | Sulfates (m eq / L) |

| | |
|---|---|
| | Magnesium (m eq / L) |
| | Sodium absorption ratio |
| B | Sand (%) |
| C | Organic matter (%) |
| | Silt (%) |
| | Soil saturation percentage |
| D | pH |
